# Diaphragmatic Endometriosis Presenting as Recurrent Catamenial Pneumothorax: A Case Report

**DOI:** 10.7759/cureus.45179

**Published:** 2023-09-13

**Authors:** Pallavi R Ganesan, Dayeon Kang, Zoya Khan, Hugh B Milteer

**Affiliations:** 1 Medical School, Alabama College of Osteopathic Medicine, Dothan, USA; 2 Internal Medicine, Mobile Infirmary Medical Center, Mobile, USA

**Keywords:** video-assisted thoracoscopic surgery (vats), thoracic endometriosis, diaphragmatic endometriosis, catamenial pneumothroax, endometriosis

## Abstract

Catamenial pneumothorax is one of the most common extra-pelvic presentations of endometriosis, with the gastrointestinal tract being the most common location. Catamenial pneumothorax is defined as spontaneous recurrent pneumothorax occurring in women of reproductive age in a temporal relationship with menses. Symptoms include dyspnea, sharp chest pain, and hypoxemia. A much rarer presentation is the involvement of endometriosis with the diaphragm.

In this case, we present a 31-year-old female who presented with signs of pneumothorax. She has had multiple episodes leading to suspicion of catamenial pneumothorax. However, it wasn’t until her surgery that the extent of diaphragmatic involvement, characterized by numerous holes secondary to endometriosis, was discovered. She was surgically treated, which led to a drastic improvement in symptoms and a reduction in subsequent episodes.

We hope that this case can add to the current limited literature on diaphragmatic endometriosis cases. Since this patient presented with mainly catamenial pneumothorax symptoms, we urge clinicians to still consider diaphragmatic involvement as a primary cause in patients with recurrent episodes of pneumothorax.

## Introduction

Endometriosis is defined as the presence of endometrial glands and stroma outside the uterine cavity, with predominance in the pelvic compartment. It is found mainly in women of reproductive age. Common symptoms include dysmenorrhea and dyspareunia. Additionally, it is also associated with infertility in some women [1].

An endometrioma is an endometrial cyst that can implant in various locations, with the most common site being the ovaries. However, an uncommon implantation site is in the thoracic region, where it can cause a constellation of symptoms most commonly referred to as thoracic endometriosis syndrome (TES) [1].

Catamenial pneumothorax is the most common presentation of TES, with diaphragmatic presentations being one of the rarer presentations. Diaphragmatic endometriosis involving the full thickness includes only 1%-1.2% of patients diagnosed with endometriosis [2]. Thoracic endometriosis syndrome is commonly surgically treated with a video-assisted thoracic surgery (VATS) procedure. Here, we present the case of a 31-year-old female who presented with signs of pneumothorax and hemoptysis and was discovered to have multiple full-thickness diaphragmatic endometriomas.

## Case presentation

We present the case of a 31-year-old African American female who presented to a local emergency department (ED) complaining of shortness of breath and hemoptysis. She had a medical history of recurrent pleural effusions for the past six months and was being followed by obstetrics and gynecology (OB-GYN) because of recurrent intra-abdominal and pelvic swelling due to a possible pelvic mass and an elevated cancer antigen-125 (CA-125) level. She was not being treated for endometriosis at this stage. She also had past episodes of hematemesis during her menses but was unable to give an accurate history of which days she specifically experienced these symptoms. Based on these symptoms, she was being monitored for possible endometriosis and was planning on undergoing a robotic or laparoscopic evaluation later that month.

Her surgical history was significant for thoracentesis and breast reduction surgery. The patient’s only medication was an over-the-counter multivitamin. The patient’s family history was unremarkable. The patient denied any smoking, tobacco use, alcohol, or illicit drug use history.

**Figure 1:**
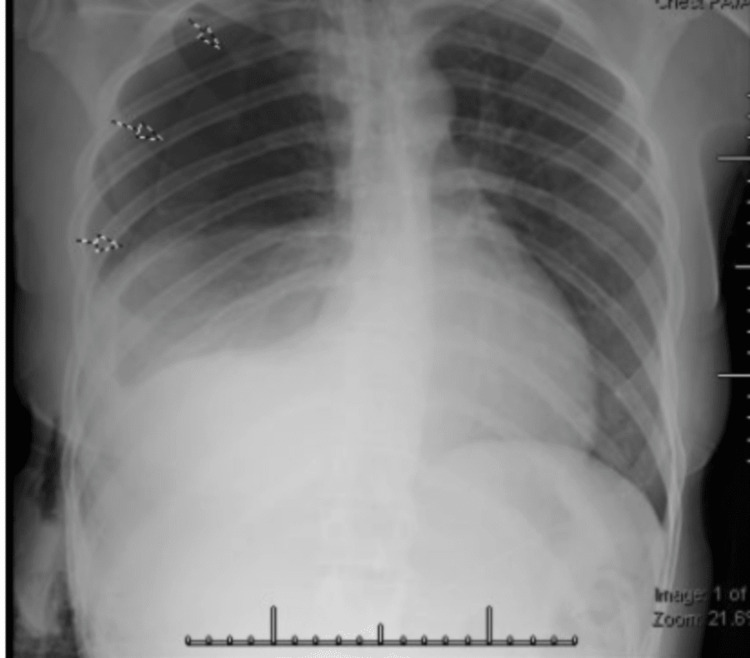
The patient's chest X-ray shows complete opacification of the right hemithorax, which is likely due to the large right pleural effusion and associated right lung atelectasis with leftward mediastinal shift.

Her vitals included a blood pressure of 136/78 mmHg, a heart rate of 78 beats per minute, a respiratory rate of 23 breaths per minute, a temperature of 98.7°F, and a pulse oximetry of 97%. A physical examination at the emergency department showed diminished breath sounds on the right side of her chest. The chest X-ray showed complete opacification of the right hemithorax, which was likely due to the large right pleural effusion and associated right lung atelectasis with leftward mediastinal shift (Figure 1).

**Figure 2:**
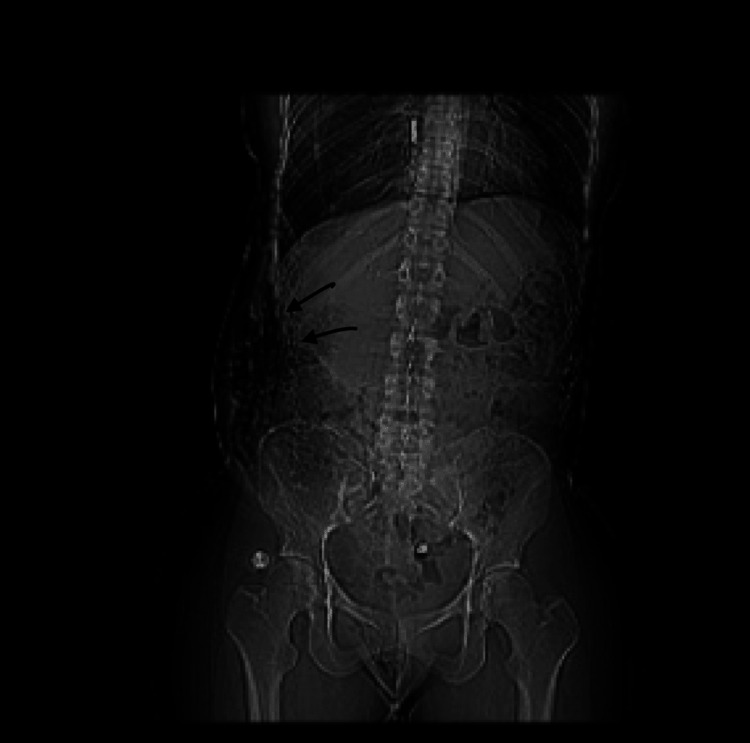
A CT of the patient's abdomen and pelvis with contrast demonstrates some ascites in the pelvis and confirms a large right-side pleural effusion with a central shift to the left side and at least two-thirds of right lung atelectasis. Compression atelectasis is visualized in the left lung.

A CT of the abdomen and pelvis with contrast demonstrated some ascites in the pelvis and confirmed large right-side pleural effusion with a central shift to the left side and at least two-thirds of right lung atelectasis. Compression atelectasis was also noted in the left lung (Figure 2).

Thoracentesis was performed, and 2 liters of bloody pleural fluid was evacuated. Based on the patient’s chronic pelvic pain, dysmenorrhea, recurrent pneumothorax, and hemoptysis, catamenial endometriosis was suspected, and further evaluation of the right thorax was needed. The patient was referred to cardiothoracic surgery to optimize her pulmonary status and confirm the diagnosis prior to pelvic intervention.

Video-assisted thoracic surgery was performed on the right side to evaluate the pleural space, evacuate the effusion, and perform biopsies. During the procedure, an additional 3.75 liters of bloody pleural fluid was evacuated, which was sent to the cytology lab. Some scarring along the edge of the diaphragm from the chronic inflammatory response as well as small pink/tan and tan-brown rubbery, irregular implants along the diaphragm were noted to be most consistent with endometriomas. Four different openings in the diaphragm that allowed fluid to freely flow from the abdomen into the right pleural space, likely causing the recurrent pleural effusions from the endometriomas, were also noted. Each opening was thoracoscopically sutured and closed. A mechanical pleurodesis was performed, including a parietal pleurectomy. The evacuated effusion was sent to microbiology and cytology labs, and several biopsies were taken and sent to pathology for analysis.

A biopsy of lesions was tested with A-calponin, Wilms' tumor suppressor gene1 (WT1), MOC21, and BerEp4 immunostains, which all supported the diagnosis of endometriosis. The cytology of the fluid showed no malignant cells and was predominantly hemosiderin-laden macrophages with scattered eosinophils. Gram stains of the fluid showed no organisms or growth. The patient was discharged after an uneventful postoperative day one.

However, the patient returned to the ED five days post-VATS surgery with worsening abdominal distention. Another right-sided tension pneumothorax with air tracking down to the right side of her abdomen was identified on a chest X-ray. This was suspected to be due to air trapped under the diaphragm after the surgery. She was treated with a chest tube, which resolved her symptoms. A decision to start the patient on intramuscular (IM) Lupron Depot, a gonadotropin-releasing hormone (GnRH), was made to prevent recurrent pneumothorax episodes and control pelvic endometriosis progression.

In the two-month post-VATS follow-up, the patient reported significantly improved breathing with no recurrent episodes of shortness of breath or hemoptysis, along with decreased pelvic pain symptoms.

## Discussion

Endometriosis is defined as the presence of endometrial glands and stroma outside the uterine cavity, with predominance in the pelvic compartment. This is an estrogen-dependent state of chronic inflammation that affects women in their reproductive period. There are multiple hypotheses about the cause of endometriosis. The pathogenic hypothesis that is supported by the most robust evidence and is the most accepted theory is based on a so-called retrograde menstruation phenomenon. This hypothesis states that some viable endometrial particles are driven through fallopian tubes by a pressure gradient caused by dyssynergic uterine contractions. Some other hypotheses include endometrial stem cell implantation, Müllerian remnant abnormalities, or coelomic metaplasia [1].

Endometriosis presents most commonly in the pelvis, with the two most common pain symptoms being dysmenorrhea and deep dyspareunia. Other symptoms include dyschezia, dysuria, infertility, and inter-menstrual pelvic pain. Diagnosis is made through exploratory laparotomy with visualization of ectopic endometrial tissue and often removal of the lesion [3].

Among the population with endometriosis, about 12% are estimated to experience endometriosis in non-reproductive organs (extragenital endometriosis). One of the more common sites of endometriosis outside the abdominopelvic cavity is in the thoracic cavity and is often referred to as thoracic endometriosis syndrome (TES) [4]. Thoracic endometriosis syndrome presents with a multitude of clinical and radiological presentations, such as catamenial pneumothorax, catamenial hemothorax, catamenial hemoptysis, and pulmonary nodules [4]. In a study by Joseph et al., they found that pelvic endometriosis was also found in 84% of women with thoracic endometriosis [5].

Catamenial pneumothorax is one of the more common presentations, occurring in about 72% of women with extra-pelvic endometriosis, and is defined as spontaneous recurrent pneumothorax that occurs in women of reproductive age, commonly in a temporal relationship with menses [5]. In patients with catamenial pneumothorax, as seen in our patient, the presenting symptoms are usually similar to those of spontaneous pneumothorax, with pleuritic chest pain, cough, and shortness of breath.

Diaphragmatic endometriosis, however, is a rarer presentation with a prevalence of 1%-1.5% of endometriosis cases [6]. It can arise independently or in association with thoracic endometriosis and is most often asymptomatic [6]. Some symptoms include hemoptysis, upper abdominal pain, painful breathing, and occasional nausea or vomiting [2]. The right side of the diaphragm is more often involved than the left diaphragm [6]. Some serious and life-threatening conditions associated with diaphragmatic endometriosis occur because of the fenestrations in the diaphragm from the necrosis of the endometriosis lesions [2].

The diagnosis of TES is based on careful intraoperative visual inspection and appropriate histological inspection of the lesions. Histology confirmed for 87.5% of patients the presence of thoracic endometriosis in the surgically treated patients [5]. The most sensitive tests for detection are chest X-rays or CT scans. An elevated CA-125 level has also been associated with endometriosis, and although it is not a specific marker, it can assist with early diagnosis [5].

The gold standard for diagnosis and treatment of TES, especially catamenial pneumothorax, is a VATS procedure [4]. In a patient with diaphragmatic endometriosis, as our patient, VATS allows for visualization of the endometriosis implants, in which hydrodissection or CO₂ laser vaporization can be performed as treatment [6]. In a study by Redwine, they found that full-thickness resection and repair of the diaphragm resulted in improvement of symptoms in 100% of patients and were curative in 88% of the population [7].

Hormonal treatment as an adjunct to surgery prevents recurrences of catamenial and/or endometriosis-related pneumothorax. Gonadotropin-releasing hormone analogs in the immediate postoperative period for six to 12 months are suggested in all patients with proven or suspected endometriosis pneumothorax [5].

In our case, the patient presented with the classic signs and symptoms of pneumothorax and recurrent hemoptysis. Her history of intra-abdominal pain due to possible mass, elevated CA-125 levels, and dyspareunia led her OB-GYN to monitor for pelvic endometriosis approximately six months prior to this presentation. There were no signs of extra-pelvic implants in CT scans or X-rays in any prior ED presentations. The top diagnoses for her symptoms were endometriosis or possible malignancy.

Thus, when she presented to our ED, our main concern with the recurrent pneumothoraces and hemoptysis was a new-onset thoracic involvement. The imaging at this presentation also showed no irregular masses that could be suspicious for endometriomas. But, due to the high degree of suspicion of extra-pelvic involvement, a VATS was performed. Involvement of the diaphragm was first identified during the procedure. The discovery of the full-thickness diaphragmatic openings and implants most likely explains her recurrent bloody pleural effusions, hemoptysis, and dyspnea.

The response to the procedure was remarkable. Although her postoperative course was only mildly complicated by abdominal distention, she reported cessation of episodes of hemoptysis, a return of her breathing to baseline, and a decrease in pelvic symptoms. She will receive IM GnRH-agonist injections for one year to control her endometriosis.

The current literature on extra-pelvic endometriosis presentations is well documented in the cases of catamenial pneumothorax. However, there were still only sporadic case reports of diaphragmatic involvement. Despite typical or atypical clinical pulmonary symptoms, there should remain a strong suspicion of thoracic endometriosis in women of childbearing age, especially if they present with concomitant pelvic signs [2]. Further research on the pathophysiology of endometriosis and its extra-pelvic presentations is warranted to better understand the variety of symptomatology and treatments.

## Conclusions

Endometriosis remains a challenge for clinicians and patients. The variations in symptoms and lack of pathophysiological understanding make it difficult to diagnose. In a woman of reproductive age who presents with hemothorax, pneumothorax, or hemoptysis, it is recommended that a diagnosis of diaphragmatic endometriosis be considered. When suspected, surgery can be indicated and often provides a treatment that has excellent outcomes. Through this report, we aim to contribute to the literature and provide another clinical presentation of diaphragmatic endometriosis.
